# Sensory properties of menthol and smoking topography

**DOI:** 10.1186/1617-9625-9-S1-S3

**Published:** 2011-05-23

**Authors:** Deirdre Lawrence, Brie Cadman, Allison C Hoffman

**Affiliations:** 1 Center for Tobacco Products, Food and Drug Administration from the National Cancer Institute, NIH, USA; 2 Freelance writer, Charlottesville, VA, USA; 3 Center for Tobacco Products, Food and Drug Administration, Rockville, MD 20850, USA

## Abstract

Although there is a great deal known about menthol as a flavoring agent in foods and confections, less is known about the particular sensory properties of menthol cigarette smoke. Similarly, although smoking topography (the unique way an individual smokes a cigarette) has been well studied using non-menthol cigarettes, there is relatively less known about how menthol affects smoking behavior. The objective of this review is to assess the sensory properties of menthol tobacco smoke, and smoking topography associated with menthol cigarettes. The cooling, analgesic, taste, and respiratory effects of menthol are well established, and studies have indicated that menthol’s sensory attributes can have an influence on the positive, or rewarding, properties associated smoking, including ratings of satisfaction, taste, perceived smoothness, and perceived irritation. Despite these sensory properties, the data regarding menthol’s effect on smoking topography are inconsistent. Many of the topography studies have limitations due to various methodological issues.

## Introduction

Menthol has a long history of safe use in mint-flavored foods and confections and has been used for lay medicinal purposes (primarily for its “cooling” effect on thermoregulation) for centuries [1]. When added to tobacco as a characterizing flavor, menthol’s cooling and anesthetic properties have been postulated to mask nicotine’s irritation, reduce harshness and soothe the taste, and add flavor to smoke, resulting in larger puffs, deeper inhalation, or longer retention time in the lungs; flavor may play an important role in initiation and sensory stimulation [2]. Determining menthol’s impact on smoking behavior can give clues to the mainstream smoke yields of multiple smoke components (e.g., tar, nicotine) and smokers’ exposure to harmful compounds. Therefore, the following questions are addressed in this paper:

• What are the sensory properties of menthol cigarettes?

• Does menthol change the way people smoke cigarettes (smoking topography)?

## Methods

Summarized in this chapter are 45 articles found to have either direct relevance to these questions, or were used to provide relevant background information. Many of these articles were identified through a review of the literature conducted by the National Cancer Institute in 2009, published as “Bibliography of literature on menthol and tobacco” http://cancercontrol.cancer.gov/tcrb/documents/menthol_bibliography_508.pdf. Additional searches and sources, such as those identified through review articles, identified additional articles that were included as appropriate. A publication or study is identified as having a tobacco industry association if one or more authors were employees of the tobacco industry, as identified by author affiliation on the publication, however no such studies are cited in the current paper.

### Sensory properties

#### Cooling and analgesia

When applied at low concentrations to skin or mucosal surfaces, menthol produces a cooling sensation [3,4]. By stimulating cold receptors found in trigeminal and dorsal root ganglia, this sensation occurs without a concomitant change in temperature [1]. The mechanism of action for both menthol and cold is activation of an excitatory calcium channel expressed by sensory neurons, the transient receptor potential melastatin-8 (TRPM8) [5] (sometimes referred to as the cold and menthol receptor or CMR1). [6] Menthol exerts cooling sensations through its agonist activation of this receptor. Menthol also stimulates the warm-activated transient receptor potential vanilloid channel 3 (TRPV3) thermoreceptor and inhibits the noxious, heat-activated transient receptor potential cation channel, subfamily A, member 1 (TRPA1) thermoreceptor (sometimes referred to as mustard oil-sensitive receptor [1,7]. In the oral cavity, menthol enhances cold sensations, but can either enhance or attenuate feelings of warmth [8].

In high concentrations, menthol can produce a burning or irritating sensation [3]. Menthol may activate pain-receptors (nociceptors) expressing TRPM8, causing these hot or irritating sensations [9]. The ability of menthol to stimulate or irritate the nerves in the mouth and throat contribute to its impact, a key component of the perceived strength of a cigarette [10]. Dessirier et al [5] showed that menthol is self-desensitizing, meaning that it has a prolonged reduction in subsequent menthol-evoked irritation. Successive administration of 0.3% menthol on the tongue resulted in progressively decreasing intensity of menthol irritation. Furthermore, menthol desensitized the tongue to nicotine, reducing nicotine’s irritating effects. The authors concluded that menthol’s cross-desensitization and potential cooling effects would reduce the degree of nicotine-induced irritation. This could enhance the acceptance of tobacco products and result in a subsequent increase in consumption of mentholated tobacco products [3].

In addition to self-desensitization, menthol cross-desensitizes a class of capsaicin-sensitive nociceptors, resulting in analgesic activity. In rodents, menthol produced dose-dependent analgesic effects, postulated to work via activation of the κ opioid system [9]. Activation of TRPM8 or inhibition of the pain-sensitive TRP1 may mediate the analgesia caused by menthol, as might its direct stimulation of cold fibers [1,12]. Topically, menthol has been shown to have anesthetic effects, and it is used in a variety of dermal pain relief products. In high concentrations, menthol produces a warming sensation, which can relieve pain similar to that of heat therapy [1]. As pain and itching are both mediated by C-fibers, menthol has been shown to have both anti-itch and anti-irritant properties [1].

#### Ease of respiration

Menthol stimulates laryngeal cold receptors [13] and nasal cold receptors in animals [14]. Menthol is often used as a decongestant and gives users the sensation of increased nasal airflow without any physical decongestant activity [15]. In four different studies, subjects reported a subjective increased sensation of airflow after exposure to menthol vapor or lozenges, even though no physical changes occurred [4,15-17]. Similarly, Nishino et al [18] found that nasal inhalation of menthol reduces the sensation of respiratory discomfort associated with loaded breathing (increased breath resistance) in a study of 11 subjects. Stimulation of cold receptors in the airway is thought be the mechanism of action for these perceived sensations of nasal decongestion and respiratory ease [18].

In vivo experiments indicate that inhalation of menthol can inhibit ventilation in guinea pigs [19] and in newborn dogs [20]. In humans, ingestion of a menthol lozenge significantly increased time between breath intake and exhalation or breath hold time [21]. In addition, menthol reduced bronchoconstriction in guinea pigs by relaxing preconstricted bronchi in vitro [22] and inhibited airway smooth muscle contraction in vivo [23]. By inhibiting respiratory rates and increasing the perception of airflow, it has been postulated that menthol may alter inhalation patterns during smoking [10]. Menthol has also been shown to be an effective antitussive (cough-suppressant) in animals [24] and humans [17]. At low concentrations, the inhalation of menthol facilitates expectoration [4].

#### Taste and smell of menthol

Menthol gives plants of the Mentha species their typical minty aroma and flavor [8]. In addition to the mint flavor, menthol can produce a bitter taste when applied to certain areas of the tongue, similar to capsaicin [25]. Menthol-flavored chewing gum elicited higher amounts of saliva compared with nicotine-containing gum and placebo gum in a study of 25 nonsmokers [26]. Similarly, in a cross-sectional study with 161 participants, menthol smokers reported an increased feeling of “wetness” in the mouth with menthol cigarettes; the researchers hypothesized that increased saliva may facilitate dissolution and absorption of nicotine in the mouth [27].

In summary, the sensory and physiologic impacts of menthol as it is inhaled are complex. As menthol and nicotine are inhaled together, both will activate sensory nerve endings in the oral, nasal, and pulmonary mucosae [3]. Menthol stimulates thermoreceptors and nociceptors, as well as olfactory, taste and trigeminal sensory receptors [8]. The varying effects of menthol—cooling and warming, irritating and analgesic, depressed ventilation and ease of inhalation—depend on concentration and formulation, duration of exposure, and temporal factors.

#### Sensory qualities associated with smoking menthol cigarettes

Consistent with menthol’s cooling, respiratory, and anesthetic effects, smokers indicate that menthol cigarettes reduce irritating effects, have a soothing effect on the lungs, and are less harsh and smoother than non-menthol cigarettes [2]. Taste and flavor are also given as reasons for smoking menthol cigarettes [2,28]. Levin et al [29] analyzed how chemosensory flavor cues affect preference in a group of menthol and non-menthol smokers. Five different types of flavors were added to a cigarette substitute, two menthol-like and three tobacco-like. The addition of flavors, especially mint, increased the acceptability of cigarette substitutes. In addition, both types of smokers liked the menthol flavors more than the placebo. The authors hypothesized that flavor and cooling sensations may provide a substitute for the tracheal stimulation involved in cigarette satisfaction [29].

Although it is difficult to assess just how menthol’s physiologic effects play into a smoker’s experience, two studies that reviewed publicly available internal tobacco industry documents indicate that menthol’s properties are used to alter numerous aspects of smoking [10,30]. Publicly available internal tobacco industry documents indicate the anesthetic properties of menthol are used to reduce pain sensations, mask the irritation of smoke, and enable initiation and increase uptake [10]. Menthol’s cooling properties help alleviate the harshness associated with smoking and allow it to function as a “smoke-soothing” agent [10]. Menthol is used to stimulate cold receptors, increase the “bite” (or strength) of smoking, and contribute to the sensory perceptions of smoke in the mouth and throat [10]. According to a review of publicly available tobacco industry documents, research associated with the tobacco industry found that menthol or mint-like flavor appears to be a main contributor to why some smokers choose menthol cigarettes [30]. Focus groups conducted by the tobacco industry found that non-menthol and menthol smokers define smoothness differently; non-menthol smokers look for absence of throat impact and tobacco taste, whereas menthol smokers define smoothness by absence of throat irritation and bitter flavor [30]. According to the authors, tobacco researchers identified menthol’s ability to increase the sensation of airflow and inhibit respiratory rates, which was used to enable deeper inhalation and alter inhalation patterns [10].

### Smoking topography

A total of eight studies contained information on smoking topography, including research on puff volume, puffs per cigarette, and other topography measures.

#### Puff volume

Seven studies have quantitatively investigated differences in puff volume with relation to cigarettes type or menthol smoker status. Although it has been postulated that mentholation of cigarettes would allow larger puff volumes, of the seven studies, three of the studies discussed found that menthol cigarettes were associated with decreased puff volume (see Table 1). Two studies failed to find any association between menthol cigarettes and puff volume, and one found that menthol cigarettes was associated with an increased puff volume.

**Table 1:** Smoking topography studies comparing puff volume among menthol and non-menthol cigarette smokers.

Menthol significantly decreased puff volume	Menthol did not produce a significant effect in puff volume	Menthol significantly increased puff volume
Jarvik et al 1994 [33] McCarthy et al 1995 [34] Nil and Battig 1989 [37]	Ahijevych et al 1996 [31] Miller et al 1995 [35] Strasser et al 2007 [36]	Ahijevych and Parsley 1999 [32]

Two studies [31,32] recruited women from the community and used a 2-factor factorial design stratified by ethnicity (Black or White) and menthol/non-menthol preference. Both of these studies allowed women to smoke their usual brand of cigarette ad lib while a flowmeter cigarette holder attached to a differential pressure transducer measured puffing topography. In the first study with 37 participants, women were equally stratified by ethnicity and menthol-smoking status. Contrary to the authors’ hypothesis, puff volume was somewhat higher for non-menthol smokers than it was for menthol smokers (48.5 versus 42.7 ml), but the difference did not reach statistical significance. The second study of 95 women using the same parameters (allowed to smoke brand of preference ad lib; equally stratified by race and menthol status) found that menthol smokers had significantly larger puff volumes compared with non-menthol smokers (45.8 versus 37.8 ml, p = .03) [32]. Because these two studies allowed women to smoke their usual brand of cigarette, it is unclear how nicotine and tar yields may have affected topography measurements between the menthol and non-menthol groups since these yields were not controlled for.

Three small, experimental cross-sectional studies involved male participants only. Jarvik et al [33] recruited 10 Black and 10 White men from the Veterans Administration Medical Center; half of the participants in each group smoked primarily menthol cigarettes. Study participants had two sessions, one week apart. In the first session, they smoked a commercially available non-menthol cigarette; in the second, they smoked a commercially available menthol cigarette. The test cigarettes had equivalent nicotine, tar, and carbon monoxide levels. Subjects smoked cigarettes as they normally would while topography measurements were taken on a smoking apparatus fitted with a Fleisch pneumotachygraph. Smoking menthol cigarettes resulted in smaller mean puff volume than the smoking non-menthol cigarettes (p < .0001), but there was no difference in the puff volumes of menthol versus non-menthol smokers. Thus, these differences appear to be due to smoker, rather than cigarette, characteristics.

A similar study recruited 29 men (11 menthol smokers and 18 non-menthol smokers) from an inpatient drug and alcohol treatment center [34]. In this within-subjects study, the subjects had two smoking sessions, one week apart, and were randomly assigned to smoke either the menthol or non-menthol cigarette first. The test cigarettes were commercially available brands chosen for their similar yields of tar, nicotine and carbon monoxide. Participants smoked the cigarettes under a rapid-smoking procedure, taking puffs every 15 seconds. The smoker could determine the size of the puff, but maximum volume was 100 cc (only one subject consistently reached this point). Topography measurements were taken in a similar apparatus to Jarvik et al [33]. The mean puff volumes were lower when participants smoked menthol cigarettes than when they smoked non-menthol cigarettes (59.64 vs. 67.15 cc; difference -7.51; 95% CI = -12.57, -2.33). Because of the unusual smoking regimen (puff every 15 seconds), and because the study population was drawn from a treatment center, it may be difficult to generalize these findings.

A study by Miller et al [35] used a similar protocol, but with investigator-applied menthol (one of three doses) to the test cigarettes. Subjects were recruited from an in-patient substance abuse ward. Twelve Black men (six menthol smokers and six non-menthol smokers) were recruited. Non-menthol Marlboro cigarettes were injected with 40 µl of alcohol-based solution that imparted 0, 4, or 8 mg of menthol. Subjects took part in three trials, 1 week apart, with each trial testing a different level of menthol. Using a smoking apparatus, subjects took puffs every 30 seconds (puff volume was set at a maximum of 100 cc). All the smokers took in a cumulative 1,200 cc of smoke before they stopped. The order in which the 12 subjects smoked the menthol levels was counterbalanced by menthol dose to control for order effects. Menthol and non-menthol smokers did not vary in the volume of the puffs they took, F (1, 10) = 0.74, ns. Menthol dosage also did not influence the volume of puffs, F (2, 9) = 0.95, ns [35].

A racially diverse pool of 116 male and female smokers participated in a smoking topography study by Strasser and colleagues [36]. Smoking topography data were collected while subjects smoked their usual brand of cigarette. Menthol smokers were not significantly different from non-menthol smokers when comparing total puff volume or mean puff volume [36].

Finally, Nil and Battig [37] recruited fifteen smokers (11 women and four men) from a Swiss population. During six different sessions, the subjects smoked six different cigarettes: high (0.8 mg) and low (0.3–0.5 mg) nicotine content cigarettes in each of three taste categories—menthol, dark, or blond tobacco (two common types of tobacco used in European cigarettes). Puff volume and puff interval were measured under natural smoking conditions and also under forced conditions, in which participants took 30 puffs with a new half-length cigarette presented after every third puff. Usual brand cigarettes were smoked in the last session as a reference. Under natural conditions, puff volume per cigarette was higher in low-yield cigarettes compared with high-yield cigarettes, as is expected due to compensatory smoking. However, the difference only reached significance in the mentholated category (p < .05). Puff volume was greater in both high- and low-yield blond tobacco compared with high-yield menthol tobacco. Under forced conditions, menthol cigarettes had the lowest puff volume as compared to dark or blonde tobacco cigarettes (p < .05) [37].

There were many methodological differences that may impact generalizability of these findings, including small study sizes, use of only men or only women in a study, differences in study design with regard to smoking as usual (ad libitum) smoking vs. rapid-smoking, and differing yield and menthol content of the cigarettes used in the study. These methodological differences make it difficult to make comparisons and draw firm conclusions.

#### Puffs per cigarette

Seven studies measured the effect of mentholation on number of puffs per cigarette (puff frequency) and the data for number of puffs per menthol cigarette vs. non-menthol cigarette are mixed (see Table 2). Jarvik et al [33] found that subjects took a smaller number of puffs from menthol cigarettes (p < .05), and that cumulative puff volume (number of puffs multiplied by volume of puffs) was smaller for smoking menthol cigarettes than it was for smoking non-menthol cigarettes (p < .001). McCarthy et al [34] found that fewer puffs were taken when smoking menthol compared with non-menthol cigarettes, and that mean aggregated volume (mean number of puffs multiplied by mean puff volume) was significantly lower for menthol cigarettes (p < .001). Also, Nil and Battig [37] showed that the high-yield menthol cigarette was smoked with significantly fewer puffs per cigarette than all the other cigarettes, with the exception of the dark, high-yield cigarette (p < .05).

**Table 2:** Published research studies comparing number of puffs per cigarette among menthol and non-menthol cigarette smokers

Significantly Fewer Puffs per Menthol Cigarette	No Statistically Significant Difference in Number of Puffs per Menthol Cigarette
Jarvik et al 1994 [33] McCarthy et al 1995 [34] Nil and Battig 1989 [37]	Ahijevych et al 1996 [31] Caskey et al 1993 [38] Miller et al 1995 [35] Strasser et al 2007 [36]

However, a study of 37 women that compared type of smoker (menthol versus non-menthol) found no difference between their number of puffs per cigarette [31]. Similarly, the dose-controlled trial by Miller et al [35] found that the number of puffs did not differ between menthol and non-menthol smokers and menthol dosage did not influence number of puffs for either type of smoker [35]. Strasser et al [36] also failed to find any menthol versus non-menthol differences in number of puffs when menthol smokers were compared with non-menthol smokers.

A seventh study by Caskey and colleagues [38] recruited 28 men from an inpatient drug and alcohol treatment center, including in 12 menthol smokers (nine Black, three White) and 16 non-menthol cigarette smokers (eight Black, eight White). Subjects participated in two rapid-smoking trials, either with a non-menthol or menthol cigarette, one week apart. Smoke was manually withdrawn from the cigarette via a syringe, and the smoker inhaled 40 cc of this smoke every 15 seconds until he or she could no longer continue. Although the researchers hypothesized that menthol cigarettes would allow smokers to take more puffs, no difference was found in the mean number of puffs between the two cigarette types; however, the authors concluded they did not have enough power to detect even a large effect size because of their small sample size. Additionally, using regimens different from smokers’ usual regimens and subjects who were in-patient drug and alcohol treatment center patients may also affect generalizability [38].

In summary, three studies show menthol cigarette smokers taking significantly fewer puffs, while four studies showing no significant difference in number of puffs between menthol and non-menthol cigarettes.

As with the studies of puff volume, there are several methodological weaknesses, including small study sizes, use of only men or only women in a study, differences in study design with regard to smoking as usual (ad libitum) vs. rapid-smoking, and differing cigarette nicotine yields and menthol content. Table 3 shows the study methodologies that were discussed in the puff volume and puff frequency sections.

The differences in study methodologies may limit the generalizability of any particular study’s findings and also make it difficult to compare results and draw firm conclusions.

**Table 3:** Methods used in published studies comparing topography measures amongst menthol and non-menthol cigarette smokers

Study	Participants	Methods
Ahijevych et al 1996 [31] Ahijevych and Parsley 1999 [32]	Women only	Both: Ad lib; smoked preferred brand of commercially available cigarettes

Caskey et al 1993 [38] Jarvik et al 1994 [33] McCarthy et al 1995 [34] Miller et al 1994 [35]	Men only	Three rapid-smoking; Jarvik ad lib; All used commercially available cigarettes

Nil and Battig 1989 [37]	Men and women	Ad lib and rapid smoking; Used low and high-yield Swiss cigarettes

Strasser et al 2007 [36]	Men and women	Ad lib; smoked preferred brand of commercially available cigarettes

#### Other topography measures

Three of the studies looked at topography parameters other than puff volume and puff frequency. Jarvik et al [33] found that mean puff flow rate was significantly lower during menthol cigarette smoking than non-menthol smoking (p < .0001), but that inhaled volume, peak puff flow, puff duration, interpuff interval, and lung retention time did not differ between menthol and non-menthol cigarettes. Similarly, Ahijevych and Parsley [32] found no significant differences in mean puff duration, mean interpuff interval, or total puff duration between menthol and non-menthol smokers. Nil and Battig [37] found that under natural smoking conditions, postpuff inspiratory times (the time over which the tidal volume is delivered following a cigarette puff) significantly increased across the taste categories from menthol cigarettes to dark, and blond tobacco cigarettes (p < .01) [37].

One study measured the potential confounding effect of race/ethnicity on topography measures in a group of menthol-only smokers. Moolchan et al [38] looked at puff volume, puff velocity, puff duration, and CO boost (increase in exhaled CO following smoke inhalation) in a group of 128 adolescent Black and White menthol smokers. Topography measures and CO boost did not differ between the groups.

#### Self-reported topography

Although topography is generally measured with quantitative measurements, three studies asked qualitative questions regarding menthol cigarette use. In a prospective cohort study of 29,037 smokers, menthol smokers’ self-reported inhalation patterns were compared with those of non-menthol smokers. Menthol and non-menthol smokers reported similar puff frequencies, depths of inhalation, and length of cigarettes smoked [40]. A baseline questionnaire given to 473 smokers in a cessation trial, however, found that menthol smokers believed menthol cigarettes to be more soothing to the throat than non-menthol cigarettes, and they felt they could inhale menthol cigarette smoke easier and deeper than smoke from non-menthol cigarettes [28]. In addition to measuring topography, Jarvik et al [33] found that subjective ratings of harshness did not differ between cigarette type (menthol or on-menthol) or between menthol and non-menthol smokers. Nil and Battig [37] found that smokers rated dark tobacco as harsher than menthol or blond tobacco (p < .0001). They also found that higher smoke yield cigarettes were rated as harsher than low-yield cigarettes (p < .001).

### Topography, menthol content and smoke yields

This section focuses specifically on menthol, nicotine, and tar yields as they relate to smoking topography.

### Menthol content/yields and cigarette preference

Three studies have looked at the effect of menthol content or smoke yield on smoking topography or cigarette preference. Miller et al [35] varied menthol content but found no effect on topography. Nil and Battig [37] found independent effects of smoke taste and smoke yield (nicotine, smoke condensate, CO) on puff volume and puff inhalation time. Increased smoking intensity, as measured by puff volume and puff inspiration time, was associated with menthol cigarettes, whereas low smoke-yield cigarettes were associated with increased puff volume, but not changes in puff inspiration time. The authors concluded that inhalation behavior is more sensitive to changes in smoke taste than to changes in smoke yield values, including those for nicotine, smoke condensate and CO [37]. A third experimental study by Pickworth et al [39] recruited 18 menthol and 18 non-menthol smokers. Each group smoked a low nicotine yield research cigarette with 0.2 mg nicotine, a commercial cigarette with 1.2 mg nicotine, and a high nicotine yield research cigarette with 2.5 mg nicotine. Cigarettes were either menthol or non-menthol, depending on the smoker’s usual preference. There was a significant difference in number of puffs per cigarettes for both groups; smokers took an average of 8.4, 11.9, and 12.8 puffs for the commercial, high nicotine yield, and low nicotine yield cigarettes, respectively (p < .01). Smokers also smoked the commercial cigarettes faster than high nicotine and low nicotine yield cigarettes (p < .01), regardless of whether or not they were menthol or non-menthol cigarettes. The authors concluded that the topography measures in this study were dependent on differences between the commercial and research cigarettes, rather than nicotine or menthol levels [41].

## Discussion

Studies have indicated that menthol’s sensory attributes can have a major influence on the positive, or rewarding, properties associated with smoking, including ratings of satisfaction, taste, perceived smoothness, and perceived irritation [2,28,45]. Focus groups and qualitative studies show that smokers choose menthols for the flavor, perceived ease of inhalation, and smoothness [30]. The cooling and analgesic effects of menthol are well established [1,8,46] and menthol’s taste and respiratory effects have been published in the literature [47]. Because of these properties, many researchers have hypothesized that menthol may affect smoking topography by way of increased breath holding, larger inhaled volume, decreased irritation, or increased palatability, thus leading to greater exposure to smoke toxins and increased nicotine dependence or disease [2,28,42]. The studies presented in this review, however, do not give a clear picture as to how menthol affects smoking topography. With regard to puff frequency, mentholated cigarettes appear to decrease frequency or have no effect, whereas as the puff volume and exhaled carbon monoxide results are conflicting or contradictory.

As noted, there are significant limitations to the topography studies in the literature. Study size, design, and methodology differences hinder comparisons and the ability to draw firm conclusions [2]. Of the seven studies reviewed, all had less than 100 participants and four had less than 20 participants. Given the large variation in how people smoke, larger sample sizes may be necessary to demonstrate significant differences. Furthermore, three of the studies [34,35,38] drew their populations from drug and alcohol treatment centers, thereby limiting external validity and the ability to generalize their findings. Four focused on men only [33-35,38] and two focused on women only [31,32]. Five studies had participants smoke menthol and non-menthol cigarettes, while the other studies measured differences between groups (menthol and non-menthol smokers). Studies also differed with regard to how the participants smoked, i.e., ad lib or under rapid smoking protocols. Puff volume and number of puffs per cigarette were the most frequently measured topography parameters, and other parameters, such as length or depth of inhalation or increased breath holding, were not routinely measured. Methodological/logistical problems with regard to measuring some variables, such as inhalation time, were noted in several studies.

While most observational and experimental studies using commercially available menthol cigarettes employed dichotomous descriptors (i.e., menthol or non-menthol), only two studies assessed menthol levels across a range of brands [30,43]. Their findings indicate that menthol levels have varied over the years and also vary significantly by sub-brand (ultralight, light, mild, non-menthol). The effects of these changes in menthol content on topography results are not clear. Specifications of menthol levels and nicotine yields would help determine which factors affect topography, since menthol may be used to offset reductions in smoke delivery or “impact” (cigarette smoke strength) in low “tar” yield cigarettes [43].

## Summary

The reviewed research may not allow firm conclusions about menthol’s role in smoking topography, but evidence from the tobacco industry documents indicates menthol is a prime player in sensory stimulation and cigarette preference [10]. According to a review of publicly available internal tobacco industry documents, the tobacco industry altered menthol content to ease the harshness of smoking, alter impact, and mask the flavor of tobacco [30]. Although publicly available internal tobacco industry documents provide a wealth of information, they often do not provide numeric data, limiting their utility.

In short, the extant literature does not bridge the gap between what is known about menthol’s multifaceted sensory effects and the mechanism by which menthol may alter a smoker’s behavior.

## Competing interests

The authors declare that they have no competing interests.
